# Hypertensive cerebral hemorrhage with undetectable plasma vascular endothelial growth factor levels in a patient receiving intravitreal injection of aflibercept for bilateral diabetic macular edema: a case report

**DOI:** 10.1186/s13256-021-02983-3

**Published:** 2021-07-27

**Authors:** Miwako Yoshimoto, Nobuhiko Takeda, Takayuki Yoshimoto, Shun Matsumoto

**Affiliations:** 1 Department of Ophthalmology, Tokyo Teishin Hospital, 2-14-23 Fujimi, Chiyoda-ku, Tokyo, 102-8798 Japan; 2 Department of Neurosurgery, Tokyo Takanawa Hospital, Tokyo, Japan; 3 Department of Immunoregulation, Institute of Medical Science, Tokyo Medical University, Tokyo, Japan

**Keywords:** Anti-vascular endothelial growth factor (VEGF) therapy, Plasma VEGF level, Diabetic macular edema, Hypertension, Cerebral hemorrhage

## Abstract

**Background:**

Intravitreal injections of anti-vascular endothelial growth factor are commonly used to treat macular diseases, including diabetic macular edema. Anti-vascular endothelial growth factor drugs can enter the systemic circulation after intravitreal injections and appear to suppress circulating vascular endothelial growth factor levels. However, whether this can cause any systemic adverse events remains unknown.

**Case presentation:**

A 70-year-old Japanese man diagnosed with diabetic macular edema in both eyes was treated with anti-vascular endothelial growth factor intravitreal injections. One month after receiving two intravitreal injections of aflibercept 1 week apart for diabetic macular edema in both eyes, he complained of a severe acute headache. The patient was diagnosed with hypertensive cerebral hemorrhage of the occipital lobe based on an elevated blood pressure of 195/108 mmHg and the results of computed tomography and magnetic resonance imaging of his brain. The patient was treated with an intravenous injection of nicardipine hydrochloride to lower his systemic blood pressure. Two days after the stroke, the patient began oral treatment with 80 mg/day telmisartan, which was continued for 3 days, and the telmisartan dose was reduced to 40 mg/day thereafter. His blood pressure promptly dropped to 130/80 mmHg, and his severe headache disappeared. One year after the cerebrovascular stroke, the telmisartan was discontinued because his blood pressure stabilized at a normal level. His plasma vascular endothelial growth factor levels were measured via specific enzyme-linked immunosorbent assay before and after the intravitreal injections of aflibercept. Immediately before the injections, the vascular endothelial growth factor level was 28 pg/ml, but it rapidly fell below the detection limit within 1 week, where it remained for over 2 months. Two days before the cerebral hemorrhage, his plasma vascular endothelial growth factor level was below the detection limit, and 2 months later after the stroke, his plasma vascular endothelial growth factor level recovered to 41 pg/ml.

**Conclusion:**

This case suggests that hypertension and resultant cerebral hemorrhage can occur in patients with diabetic macular edema when plasma vascular endothelial growth factor levels are systemically decreased below the detection limit for a prolonged time after local injections of anti-vascular endothelial growth factor agents into the vitreous cavity. Therefore, severely reduced plasma vascular endothelial growth factor levels could be a higher risk factor to develop generally infrequent stroke. Ophthalmologists should be aware of possible severe reduction of plasma vascular endothelial growth factor levels and resultant increase in blood pressure after intravitreal injections of an anti-vascular endothelial growth factor drug. If the plasma vascular endothelial growth factor levels could be monitored more easily and quickly during the treatment, it would help to prevent adverse events.

## Background

Vascular endothelial growth factor (VEGF) plays an essential role in normal and abnormal vasculogenesis and angiogenesis [1]. VEGF stimulates endothelial cell proliferation, survival, and migration, which are necessary to form new blood vessels during embryonic development and wound healing [1]. Studies have shown that VEGF plays a central role in the pathogenesis of tumor growth and metastasis and in retinopathy associated with several blinding eye diseases, including diabetic macular edema (DME) [1]. The anti-VEGF monoclonal antibody, bevacizumab, was approved as a first-line treatment for metastatic colorectal cancer [2]. Anti-VEGF therapy is also widely recommended to maintain visual functioning in patients with age-related macular degeneration, retinal vein occlusion, myopic choroidal neovascularization, and DME [1, 2]. Large clinical studies [3–6] have confirmed the safety of anti-VEGF therapy, but some meta-analyses [7] indicate a high risk of systemic adverse events after repeated intravitreal anti-VEGF therapy for DME. This inconsistency may result from the study design strategy because large studies are often limited to patients with relatively fewer and minor complications and exclude patients at risk for the worst adverse events such as cerebrovascular and arteriothrombotic events [4, 8]. Because patients with DME generally exhibit systemic complications, investigating the direct adverse events of anti-VEGF therapy for DME is difficult. Blockage of the VEGF pathway in cancer patients can cause adverse events such as hypertension, arterial thromboembolic events, cardiac dysfunction, proteinuria, renal toxicity, and compromised wound healing and tissue repair [9, 10]. Previous studies demonstrated that plasma VEGF levels were suppressed after local intravitreal anti-VEGF therapy and continued to be decreased for several weeks [11, 12]. Other studies of age-related macular degeneration have reported elevated blood pressure after intravitreal anti-VEGF therapy [13, 14]. However, evidence of a direct relationship between decreased plasma VEGF levels and adverse events, such as hypertension and resultant cerebral hemorrhage, is lacking.

Here, we report a case of hypertensive cerebral hemorrhage after intravitreal injections of aflibercept for bilateral DME, in which the plasma VEGF levels remained below the detection limit before and after the stroke. We also present a comprehensive review of the relevant literature.

## Case presentation

A 70-year-old Japanese man without any major family and psychosocial history was referred to our hospital to continue treatment for DME. At the patient’s former hospital, his major symptom was repeated visual disturbance of both eyes due to DME, and he received sub-Tenon’s triamcinolone acetonide injection and intravitreal anti-VEGF injection. Sub-Tenon’s triamcinolone acetonide injections were once in both eyes, and intravitreal anti-VEGF injections were 12 times in both eyes every 2–3 months (4 times with 0.5 mg ranibizumab and 7 times with 2  mg aflibercept in the right eye and once with aflibercept in the left eye) during the 2.5-year follow-up period. At the patient’s former hospital, the last anti-VEGF therapies were injected in the left eye in July 2016 and the right eye in December 2016.

At the patient’s first visit to our hospital in January 2017, his body height and weight was 160 cm and 52 kg [body mass index (BMI) 20.3], respectively. His HbA1c level was 6.3 %, and he has been suffering from diabetes mellitus for 10 years with only diet therapy. His blood pressure was 120/61 mmHg, and he had no hypertension. Although he had hyperlipidemia (total cholesterol 244 mg/dl, LDL cholesterol 148 mg/dl), he did not receive any pharmacological treatments for it. He has been treated with donepezil hydrochloride for mild cognitive impairment in the neurology department of Tokyo Takanawa Hospital. However, because he works regularly and can attend a hospital by himself, he has no hindrance to daily life. In addition, there was no issue on financial and language/cultural challenges. A dilated fundus examination showed mild nonproliferative diabetic retinopathy [15], with several small hemorrhages without macular edema, and his best corrected visual acuity was 1.0 in both eyes. The central subfield macular thickness (CMT) on spectral-domain optical coherence tomography (SD-OCT) was 279/320 μm (right eye/left eye).

**Fig. 1:**
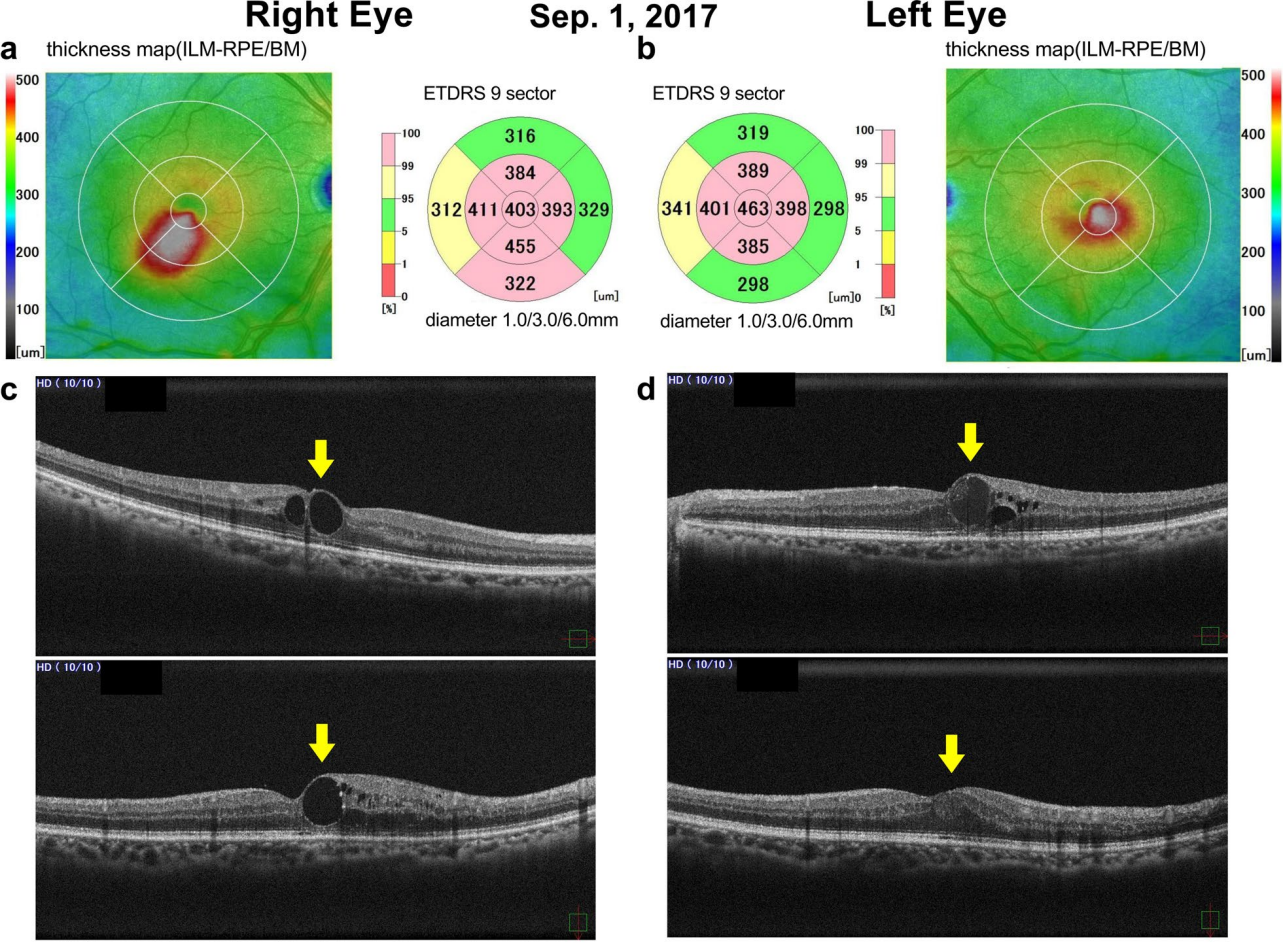
Spectral-domain optical coherence tomography findings from 1 September 2017. **a** Right-eye thickness map; **b** left-eye thickness map; **c** right-eye line scan; **d** left-eye line scan. Upper figure is the horizontal scan; lower figure is the vertical scan of the macula. Three months after anti-vascular endothelial growth factor therapy in both eyes, the concomitant binocular diabetic macular edema, relapsed. Yellow arrows indicate cystoid macular edema

**Fig. 2:**
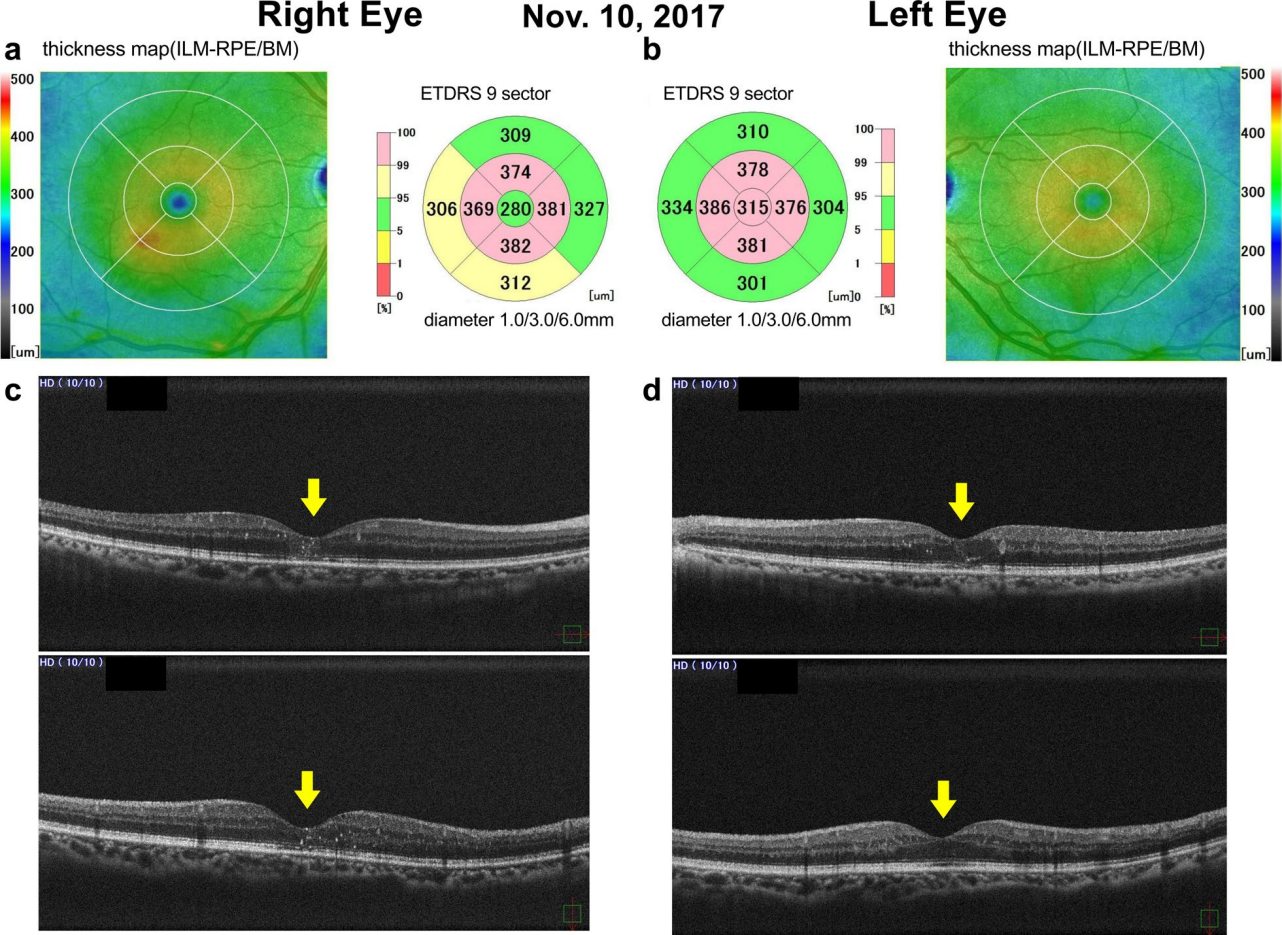
Spectral-domain optical coherence tomograph findings from 10 November 2017 (1 month after treatment). **a**–**d** are the same as in Fig. 1. Anti-vascular endothelial growth factor therapy was effective, and the binocular diabetic macular edema disappeared entirely. Yellow arrows indicate disappearance of the cystoid macular edema

Five months later, he noticed blurred vision in both eyes (visual acuity was 0.8 in both eyes), and a fundus examination showed significant macular edema in both eyes (CMT, 404/478 μm). In June 2017, for the first time at our hospital, the patient received 2-mg intravitreal aflibercept injections in both eyes 1 week apart (6 June in the right eye and 13 June in the left eye). No laser therapy was applied. After short-term anti-VEGF therapies to both eyes, his systemic condition remained the same. The treatment was effective in both eyes, and 1 month later, his CMT recovered to 277/321  μm, and his blurred vision disappeared (visual acuity was 1.0 in right eye and 1.2 in left eye). Three months after the first treatment at our hospital (1 September 2017), significant macular edema relapsed in both eyes (CMT, 403/463 μm; Fig. 1), visual acuity was 0.8 in right eye and 1.0 in left eye, and he desired continuous treatment for both eyes. In October 2017, he received intravitreal aflibercept injections in both eyes 1 week apart (3 October in the right eye and 10 October in the left eye). One month after treatment (10 November), his subjective complaint of blurred vision disappeared (0.8 in right eye and 1.2 in left eye), and the CMT decreased to 280/315 μm (Fig. 2). During this period, his HbA1c levels were measured and kept at 6.5% (3 October, 10 October, and 10 November).

**Fig. 3:**
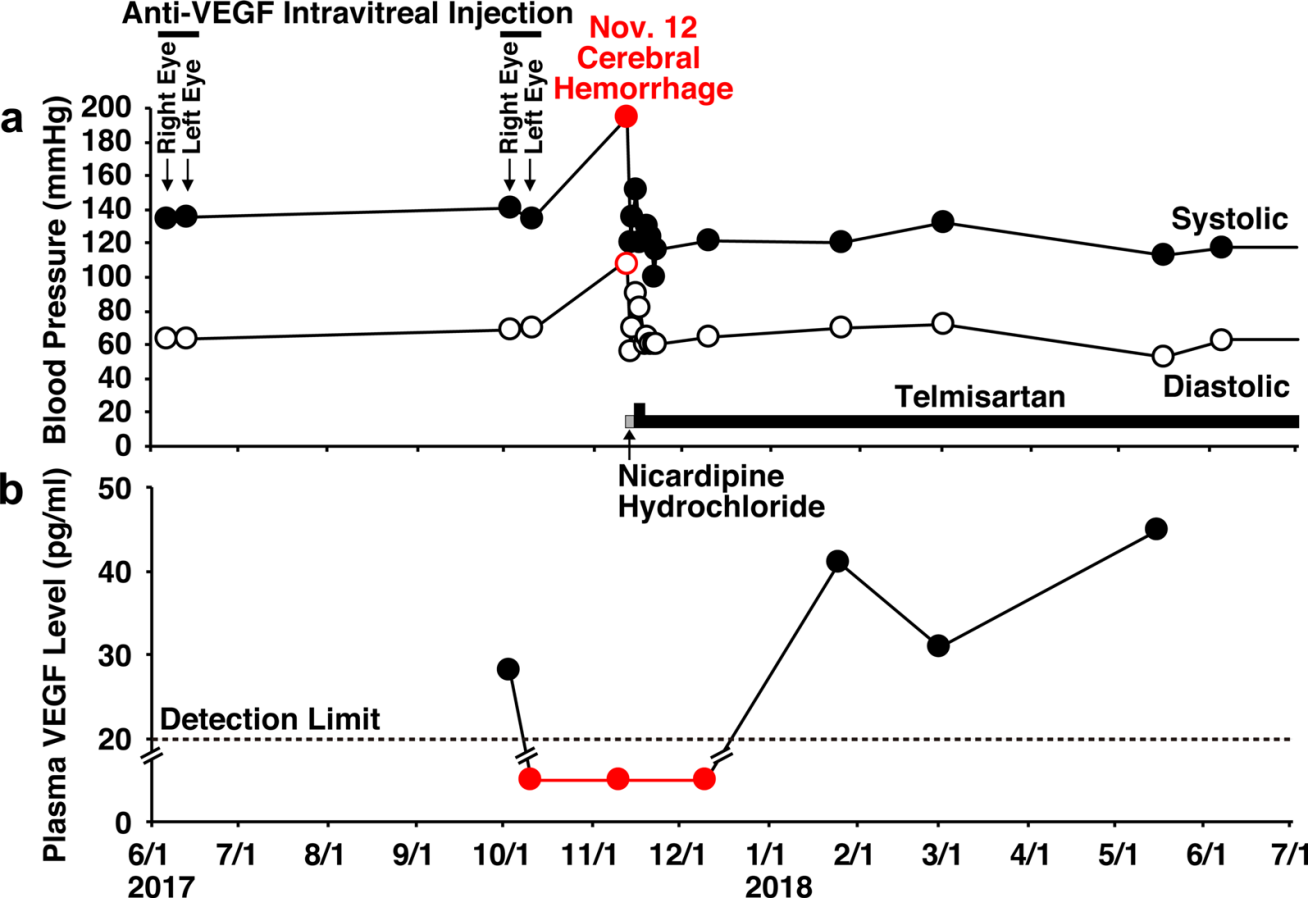
Systolic and diastolic blood pressure (**a**) and plasma vascular endothelial growth factor levels (**b**) of the patient before and after binocular anti-VEGF therapies 1 week apart. More than 2 months after anti-VEGF therapy in both eyes, the plasma VEGF levels fell below the detection limit (< 20 pg/ml). On 12 November 2017, cerebral hemorrhage occurred owing to acute hypertension, and the patient was treated with an intravenous injection of nicardipine hydrochloride for 2 days to lower his blood pressure, which promptly returned to a normal level within the same day. On 14 November, 2 days after the stroke, the patients began oral treatment with 80 mg/day telmisartan, which was continued for 3 days. Starting 17 November, the telmisartan dose was reduced to 40 mg/day and continued for 1 year

**Fig. 4:**
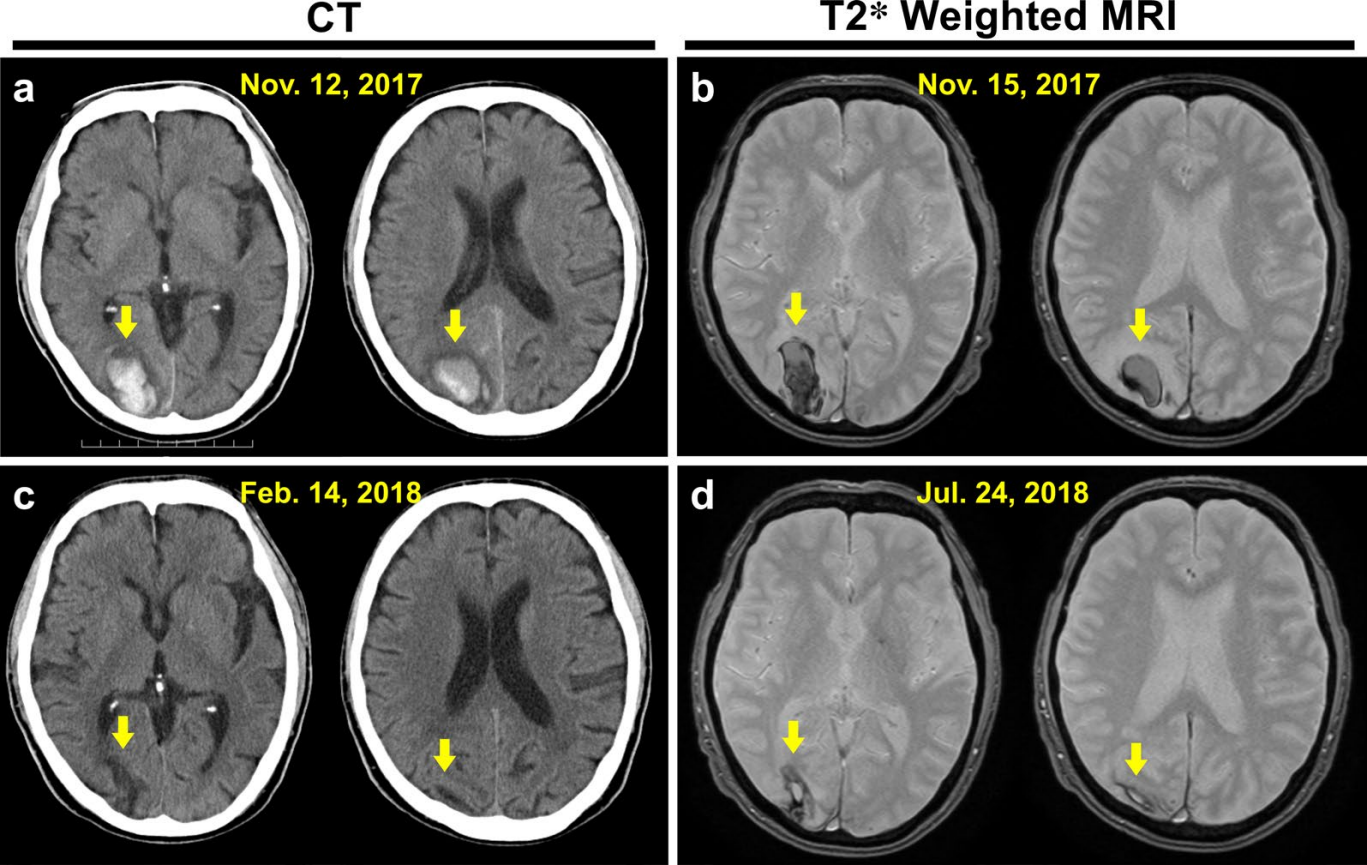
Computed tomography (CT) of the brain on 12 November 2017 (**a**) and 14 February 2018 (**c**), and T2*-weighted magnetic resonance imaging (MRI) of the brain on 15 November 2017 (**b**) and 24 July 2018 (**d**). Two different slices were included on each date. High density on the CT (**a** yellow arrowhead) and low intensity of the peripheral zone of the lesion on the T2*-weighted MRI (**b** yellow arrowhead) revealed cerebral hemorrhage of the right occipital lobe. Three months after the stroke, the high-density area disappeared, and the small low-density area remained on the brain CT (**c** yellow arrowhead). The low-intensity lesion decreased in size 8 months later on the brain T2*-weighted MRI (**d** yellow arrowheads)

**Fig. 5:**
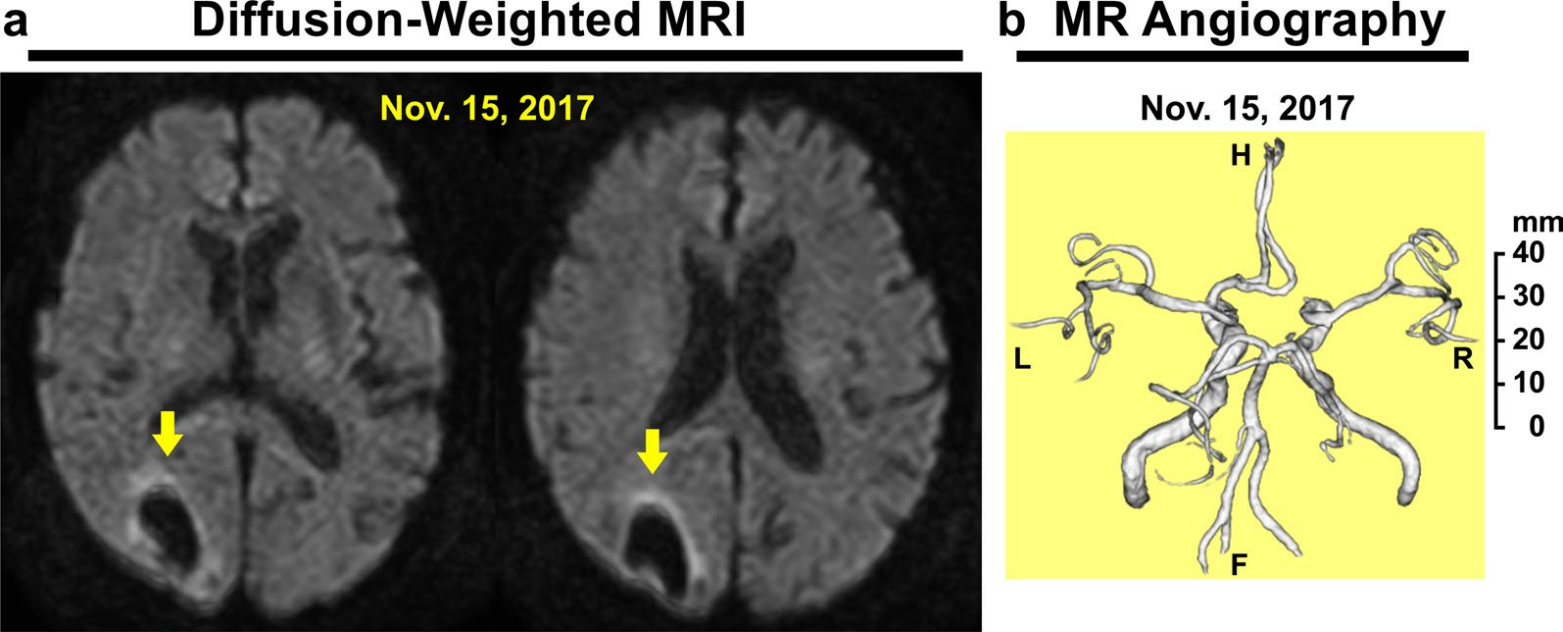
Diffusion-weighted magnetic resonance imaging (MRI) (**a**) and magnetic resonance (MR) angiography (**b**) of the brain on 15 November 2017. Two different slices were included (**a**). Diffusion-weighted MRI revealed the low-intensity area in the same region of the right occipital lobe (**a** yellow arrowheads); MR angiography demonstrated no vascular anomalies or malformation. *H* head, *F* feet, *R* right, *L* left (**b**)

**Fig. 6:**
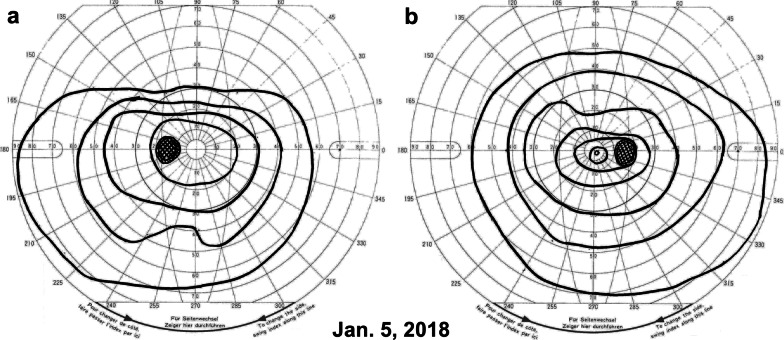
Goldmann perimetry of both eyes on 5 January 2018 (2 months after the cerebral hemorrhage). Goldmann perimetry showed no apparent visual field abnormalities. **a** left eye, **b** right eye

Upon initiating the intravitreal injection in the operating room, we measured his blood pressure as 141/69 mmHg (3 October) and 135/70 mmHg (10 October) without blood-pressure-lowering drugs (Fig. 3a). However, on 12 November, just 2 days after a follow-up visit to our hospital, he complained of a severe headache and walked to the emergency department at Tokyo Takanawa Hospital. He complained of a severe headache on the right back side of his head and visual disturbance of the left visual field with no other systemic neuropathological symptoms. He had no external injury presumed as trauma antecedent and no other apparent causes leading to cerebral hemorrhage. His blood pressure was elevated at 195/108 mmHg, and computed tomography (CT) of the brain revealed the presence of a high-density area (2.5 × 3.0 cm) in the right occipital lobe indicating a subcortical hemorrhage (Fig. 4a). The patient was treated with an intravenous injection of nicardipine hydrochloride to lower his systemic blood pressure to < 140 mmHg for 2 days. On 13 November, CT showed no enlargement of the high-density area (data not shown). On 15 November, 3 days after the stroke, magnetic resonance imaging (MRI) revealed a peripheral low-intensity zone in the same region (T2*-weighted MRI, Fig. 4b) and a low-intensity area (diffusion-weighted MRI, Fig. 5a) in the occipital lobe that was consistent with the CT results. Additionally, magnetic resonance (MR) angiography revealed no vascular anomalies or malformation (Fig. 5b), and T2*-weighted MRI revealed no cerebral microbleeds, thus ruling out cerebral amyloid angiopathy (Fig. 4b). He was therefore diagnosed with a hypertensive cerebral hemorrhage of the occipital lobe. On 14 November, 2 days after the stroke, the patient began oral treatment with 80 mg/day telmisartan, which was continued for 3 days. Starting 17 November, the telmisartan dose was reduced to 40 mg/day. During this period, his blood pressure was maintained at < 130/80 mmHg, and his symptoms of severe headache disappeared, and visual disturbance of the left visual field was alleviated. Ten days later, he was discharged from Tokyo Takanawa Hospital, and 20 days after discharge he visited our hospital to receive a follow-up ophthalmological examination. Two months after the stroke (5 January 2018), visual field tests with Goldmann perimetry at our hospital showed no apparent left homonymous hemianopsia (Fig. 6). Follow-up CT (14 February 2018; Fig. 4c) and T2*-weighted MRI (24 July 2018; Fig. 4d) examinations revealed that the cerebral hemorrhage had been absorbed, and the lesion had decreased in size. One year after the cerebrovascular stroke, the telmisartan was discontinued because his blood pressure had stabilized to a normal level and remained around 110/60 mmHg.

The patient received no anti-VEGF therapy for 4 months prior to 3 October 2017 because his macular edema did not relapse. We happened to determine his plasma VEGF levels before and after the intravitreal anti-VEGF injections (October 2017) via specific enzyme-linked immunosorbent assay (SRL, Tokyo, Japan; Fig. 3b). The patient’s plasma VEGF level was 28 pg/ml on 3 October, just before the intravitreal injection of aflibercept in the right eye. However, the level rapidly decreased to below the detection limit (< 20 pg/ml) on 10 October, just before the injection in his left eye. Notably, the levels on 10 November (2 days before the cerebral hemorrhage) and 10 December were also below the detection limit. On 25 January 2018, we confirmed that his plasma VEGF level had recovered to 41 pg/ml. Thereafter, we continued to follow the patient for DME and diabetic retinopathy, and no cerebral hemorrhage recurred.

## Discussion

This is the first report of a patient who experienced hypertensive cerebral hemorrhage when plasma VEGF levels were below detection limit before and after stroke, on a patient receiving intravitreal injection of aflibercept for bilateral DME. Decreased plasma VEGF levels are possibly suspected to have a causal relationship with hypertensive cerebral hemorrhage because VEGF is expressed in nearly all organs and tissues and is essential in normal and abnormal endothelial vasculogenesis and angiogenesis [1, 16]. VEGF induces new blood vessel formation by increasing the capillary and arteriolar density and releasing nitric oxide and prostaglandin I_2_, which inhibit platelet activation, via endothelial cells to promote vasodilation in arterioles and venules [1, 16]. In the absence of VEGF, normal blood vessel formation by endothelial cells is impaired and weakened and thus more susceptible to hypertension and resultant hemorrhage. However, clarifying a direct causal relationship is difficult because DME patients generally have systemic complications. Although several trials revealed that intravitreal injection of anti-VEGF agents does not increase the risk of systemic vascular events [3–5], uncertainty still remains for DME patients who are at high risk for vascular disease and were not included [4]. High blood pressure is thus considered to increase the risk of cerebral hemorrhage, but in this patient the most critical key incident was likely the prolonged absence of plasma VEGF together with the resultant high blood pressure due to the anti-VEGF treatment in both eyes over a short interval. To more clearly reveal a causal relationship between VEGF plasma levels and such adverse events, further rigorous clinical trials are necessary.

Plasma VEGF levels are not routinely monitored, but we investigated the plasma VEGF levels of 15 patients with DME from October 2017 to September 2018 and found that almost all patients showed decreased plasma VEGF levels after treatment with anti-VEGF agents (data not shown). Among them, two patients, including the patient described herein and a younger patient (a 45-year-old woman), received anti-VEGF agents to both eyes 1 week apart. In May 2013, the younger patient developed a cerebral infraction, and it turned out that she has also diabetes mellitus. When she started the treatment with intravitreal injections of anti-VEGF agents (ranibizumab), she was treated with teneligliptin hydrobromide hydrate, bezafibrate, and aspirin, and her HbA1c level and blood pressure was 6.4% and 119/76 mmHg, respectively. Her plasma VEGF levels fell below the detection limit 1 day after the second treatment, which was 1 week after the first treatment, and 2 weeks after the treatment her VEGF levels recovered to detectable levels (21 pg/ml, and 1 month later 22 pg/ml). However, the present patient developed hypertensive cerebral hemorrhage with severely reduced plasma VEGF levels that had been below the detection limit for more prolonged time after the second treatment with the anti-VEGF agent to both eyes than those in the younger patient. Follow-up CT and T2*-weighted MRI examinations (Fig 4b, d) suggested that the patient healed as normal because his blood pressure promptly dropped to the normal level within the same day after immediate treatment with an intravenous injection of nicardipine hydrochloride, which was maintained with oral telmisartan thereafter (Fig. 3). Consequently, the plasma VEGF level was recovered by 25 January 2018 (Fig. 3). Although this report presents only one case, clinicians should consider that adverse events, including hypertension and resultant cerebral hemorrhage, can occur if plasma VEGF levels fall below the detection limit for a prolonged time during anti-VEGF therapy for DME.

Plasma VEGF levels that systemically fall below the detection limit after local intravitreal injections of anti-VEGF agents pose risks similar to those seen in cancer patients [17]. The three most common anti-VEGF agents are bevacizumab, aflibercept, and ranibizumab. Owing to their molecular properties [11, 18, 19], bevacizumab and aflibercept were reported to greatly reduce plasma VEGF levels 1 and 4 weeks after a single intravitreal injection, whereas ranibizumab had no significant effect on these levels [12]. However, ranibizumab presented a possible risk for cerebrovascular events when used monthly for 2 years [7]. There is no difference among the treatments with these anti-VEGF agents in the risk of myocardial infraction, acute cerebrovascular disease, and major bleeding [3, 20, 21]. Importantly, intraocular VEGF levels were recently correlated with retinal neovascularization and consequently DME resolution but were unrelated to the DME severity or diabetic retinopathy [12, 22, 23]. Therefore, decreased plasma VEGF levels are unnecessary for treating DME, and the total anti-VEGF agent amounts should be minimized to avoid prolonged decreases in plasma VEGF levels and possible resultant systemic adverse events in DME patients.

Risk factors for DME are high hemoglobin A1c levels and a long duration of diabetes [24], and many patients with DME have cardiovascular and cerebrovascular complications. Intravitreal anti-VEGF therapy improves visual outcomes and decreases macular edema, but the response is more gradual than that of other retinal diseases [25]. Patients with diabetes require more intravitreal injections of anti-VEGF medications to attain maximal improvement of visual outcomes. These injections must be given more often because DME is often binocular. Thus, to treat DME, clinicians might be better to combine anti-VEGF injections with other therapies (direct photocoagulation of microaneurysms or sub-Tenon’s triamcinolone acetonide injection and vitrectomy) as well as systemic improvement of diabetes control with the inclusion of sodium–glucose cotransporter 2 inhibitors [26] and lipid metabolism. If anti-VEGF therapy must be frequently repeated, changes in plasma VEGF levels and resultant blood pressure together with other systemic pathological objective conditions should be monitored at every treatment in addition to the patient’s self-reported health status, especially in concomitant binocular treatment. Thus, severely reduced plasma VEGF levels could be another higher risk factor to develop generally infrequent stroke. Improving the technique for determining plasma VEGF levels would enable easier and faster measurements, thus making anti-VEGF therapy for DME safer. This could determine a more effective treatment schedule and may lead to better and safer vision and life quality for patients with diabetes.

## Conclusions

Hypertension and resultant cerebral hemorrhage can occur as adverse events in patients with DME when plasma VEGF levels systemically fall below the detection limit for a prolonged time after locally injecting anti-VEGF agents into the vitreous cavity. Therefore, severely reduced plasma VEGF levels could be a higher risk factor to develop generally infrequent stroke. Ophthalmologists should be aware of possible severe reduction of plasma VEGF levels and resultant increase in blood pressure after intravitreal injections of an anti-VEGF drug. If the plasma VEGF levels could be monitored more easily and quickly during the treatment, it would help to prevent adverse events.

## Data Availability

All data generated or analyzed during this study are included in this published article.

## Abbreviations

CMT: Central subfield macular thickness
CT: Computed tomography
DME: Diabetic macular edema
VEGF: Vascular endothelial growth factor
MRI: Magnetic resonance imaging
SD-OCT: Spectral-domain optical coherence tomography
